# Tetra­aqua­bis­[5-(pyridin-3-yl)tetra­zolido-κ*N* ^5^]manganese(II) tetra­hydrate

**DOI:** 10.1107/S160053681202510X

**Published:** 2012-06-13

**Authors:** Chen Qi, Xiang He, Min Shao, Ming-Xing Li

**Affiliations:** a Department of Chemistry, College of Science, Shanghai University, Shanghai, 200444, People’s Republic of China; b Laboratory for Microstructures, Shanghai University, Shanghai 200444, People’s, Republic of China

## Abstract

The title compound, [Mn(C_6_H_4_N_5_)_2_(H_2_O)_4_]·4H_2_O, was obtained by the solution reaction of MnCl_2_ and 3-(2*H*-tetra­zol-5-yl)pyridine. The Mn^II^ atom, located on an inversion center, shows a slightly distorted octa­hedral geometry and is coordinated by two pyridine N atoms from two 5-(pyridin-3-yl)tetra­zolide ligands occupying *trans* positions and four water mol­ecules. In the crystal, the mononuclear complex mol­ecules and solvent water mol­ecules are connected into a three-dimensional framework by O—H⋯N and O—H⋯O hydrogen bonds.

## Related literature

For the synthesis and crystal structure of the isotypic zinc(II) complex [Zn(C_6_H_4_N_5_)_2_(H_2_O)_4_]·4H_2_O, see: Mu *et al.* (2010 ▶).
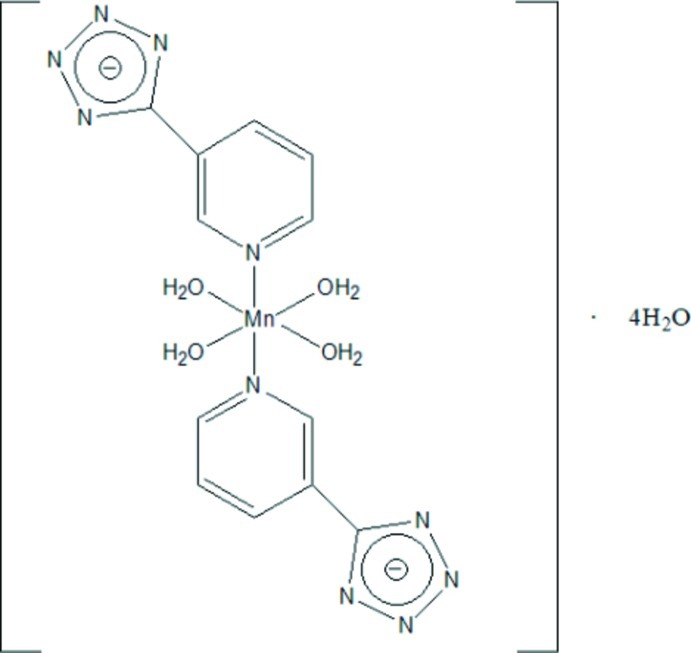


## Experimental

### 

#### Crystal data

[Mn(C_6_H_4_N_5_)_2_(H_2_O)_4_]·4H_2_O
*M* *_r_* = 491.35
Triclinic, 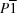

*a* = 8.137 (8) Å
*b* = 8.629 (8) Å
*c* = 8.761 (8) Å
α = 84.878 (10)°
β = 65.347 (8)°
γ = 72.571 (10)°
*V* = 533.0 (9) Å^3^
*Z* = 1
Mo *K*α radiation
μ = 0.68 mm^−1^
*T* = 293 K
0.15 × 0.10 × 0.10 mm

#### Data collection

Bruker APEXII CCD diffractometer
Absorption correction: multi-scan (*SADABS*; Sheldrick, 2007 ▶) *T*_min_ = 0.922, *T*_max_ = 0.934
2785 measured reflections
1850 independent reflections
1712 reflections with *I* > 2σ(*I*)
*R*_int_ = 0.026

#### Refinement

*R*[*F* ^2^ > 2σ(*F* ^2^)] = 0.032
*wR*(*F* ^2^) = 0.080
*S* = 1.05
1850 reflections
143 parameters
H-atom parameters constrained
Δρ_max_ = 0.23 e Å^−3^
Δρ_min_ = −0.32 e Å^−3^

### 

Data collection: *APEX2* (Bruker, 2000 ▶); cell refinement: *SAINT* (Bruker, 2000 ▶); data reduction: *SAINT*; program(s) used to solve structure: *SHELXS97* (Sheldrick, 2008 ▶); program(s) used to refine structure: *SHELXL97* (Sheldrick, 2008 ▶); molecular graphics: *SHELXTL* (Sheldrick, 2008 ▶); software used to prepare material for publication: *SHELXTL*.

## Supplementary Material

Crystal structure: contains datablock(s) I, global. DOI: 10.1107/S160053681202510X/gk2497sup1.cif

Structure factors: contains datablock(s) I. DOI: 10.1107/S160053681202510X/gk2497Isup2.hkl

Additional supplementary materials:  crystallographic information; 3D view; checkCIF report

## Figures and Tables

**Table 1:** Hydrogen-bond geometry (Å, °)

*D*—H⋯*A*	*D*—H	H⋯*A*	*D*⋯*A*	*D*—H⋯*A*
O1—H1*B*⋯O4^i^	0.85	1.94	2.783 (3)	172
O1—H1*A*⋯N5^ii^	0.85	1.91	2.731 (3)	163
O2—H2*A*⋯O3^iii^	0.85	1.99	2.836 (3)	171
O2—H2*B*⋯O3^iv^	0.85	1.96	2.800 (3)	169
O3—H3*B*⋯O4	0.85	1.96	2.803 (3)	171
O3—H3*A*⋯N2	0.85	1.96	2.797 (3)	170
O4—H4*B*⋯N3^v^	0.85	2.03	2.878 (3)	177
O4—H4*A*⋯N4^vi^	0.85	2.00	2.849 (3)	176

Symmetry codes: (i) 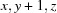; (ii) 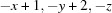; (iii) 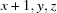; (iv) 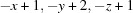; (v) 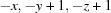; (vi) 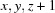.

**Fig. 1.**
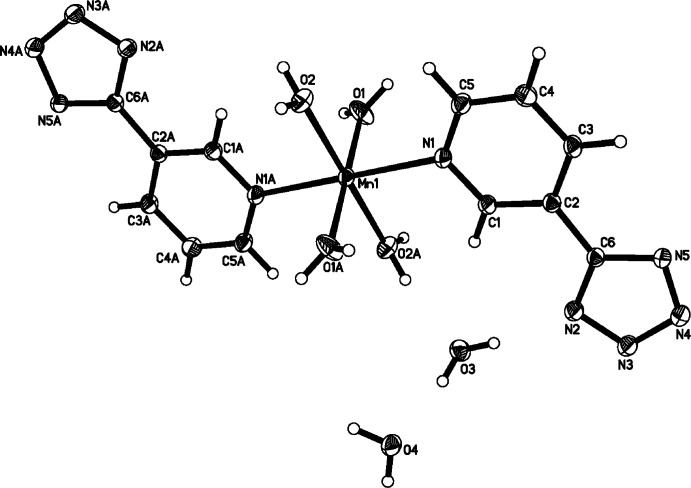
The asymmetric unit of the title complex. Displacement ellipsoids are drawn at the 30% probability level. [Symmetry code: (A) -x, -y, -z].

**Fig. 2.**
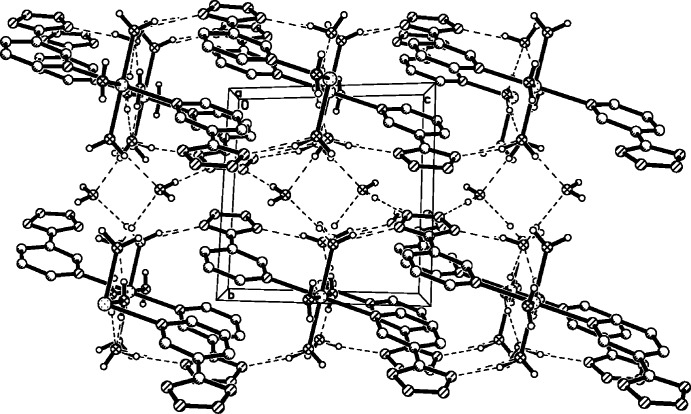
A crystal packing diagram of the title compound with hydrogen bonds shown as dashed lines. H atoms not involved in hydrogen bonding have been omitted for clarity.

## supplementary crystallographic information

### Comment

3-(2*H*-Tetrazol-5-yl)pyridine (3-Ptz) is a multifunctional ligand which possesses five potential coordinate nitrogen atoms. Recently Mu *et al.* (2010) reported that hydrothermal reaction of Zn(OAc)_2_ with 3-Ptz results in a mononuclear zinc complex [Zn(C_6_H_4_N_5_)_2_(H_2_O)_4_].4H_2_O. We were able to prepare an analogues manganese(II) compound, [Mn(C_6_H_4_N_5_)_2_(H_2_O)_4_].4H_2_O, by the solution reaction of MnCl_2_ with 3-Ptz in a basic H_2_O/ethanol solution. This compound is closely isostructural with the Zn complex reported by Mu *et al.* (2010)

### Experimental

A mixture of MnCl_2_ (0.1 mmol), 3-Ptz (0.1 mmol), 1 ml NaOH solution (0.1 mol *L*^-1^) was added into 10 ml H_2_O/ethanol mixed solvent (1:1). After being stirred for twenty minutes, the mixture was filtered. The filtrate was left undisturbed for two days to give yellow block crystals with 35% yield based on 3-Ptz. Anal. calcd for C_12_H_24_MnN_10_O_8_ (%): C, 29.33; H, 4.92; N, 28.51. Found: C, 29.24; H, 4.83; N, 28.66. IR (KBr pellet, cm^-1^): 3400*m*, 1613*m*, 1588*m*, 1464*m*, 1426s, 1372*m*, 1153s, 1019*m*, 787s, 750s, 696s, 642*m*, 463*m*.

### Refinement

All the H atoms were positioned geometrically (C—H = 0.93 Å, O—H = 0.85 Å), and allowed to ride on their parent atoms, with *U*_iso_(H) = 1.2 *U*_eq_(C) or 1.5Ueq(O).

### Crystal data

**Table:**

[Mn(C_6_H_4_N_5_)_2_(H_2_O)_4_]·4H_2_O	*Z* = 1
*M**_r_* = 491.35	*F*(000) = 255
Triclinic, *P*1	*D*_x_ = 1.531 Mg m^−^^3^
Hall symbol: -P 1	Mo *K*α radiation, λ = 0.71073 Å
*a* = 8.137 (8) Å	Cell parameters from 1565 reflections
*b* = 8.629 (8) Å	θ = 2.5–27.3°
*c* = 8.761 (8) Å	µ = 0.68 mm^−^^1^
α = 84.878 (10)°	*T* = 293 K
β = 65.347 (8)°	Block, yellow
γ = 72.571 (10)°	0.15 × 0.10 × 0.10 mm
*V* = 533.0 (9) Å^3^	

### Data collection

**Table:**

Bruker APEXII CCD diffractometer	1850 independent reflections
Radiation source: fine-focus sealed tube	1712 reflections with *I* > 2σ(*I*)
Graphite monochromator	*R*_int_ = 0.026
phi and ω scans	θ_max_ = 25.0°, θ_min_ = 2.5°
Absorption correction: multi-scan (*SADABS*; Sheldrick, 2007)	*h* = −7→9
*T*_min_ = 0.922, *T*_max_ = 0.934	*k* = −6→10
2785 measured reflections	*l* = −10→10

### Refinement

**Table:**

Refinement on *F*^2^	Secondary atom site location: difference Fourier map
Least-squares matrix: full	Hydrogen site location: inferred from neighbouring sites
*R*[*F*^2^ > 2σ(*F*^2^)] = 0.032	H-atom parameters constrained
*wR*(*F*^2^) = 0.080	*w* = 1/[σ^2^(*F*_o_^2^) + (0.0343*P*)^2^ + 0.2064*P*] where *P* = (*F*_o_^2^ + 2*F*_c_^2^)/3
*S* = 1.05	(Δ/σ)_max_ < 0.001
1850 reflections	Δρ_max_ = 0.23 e Å^−^^3^
143 parameters	Δρ_min_ = −0.32 e Å^−^^3^
0 restraints	Extinction correction: *SHELXL*, Fc^*^=kFc[1+0.001xFc^2^λ^3^/sin(2θ)]^-1/4^
Primary atom site location: structure-invariant direct methods	Extinction coefficient: 0.063 (6)

### Special details

**Table:**

Geometry. All e.s.d.'s (except the e.s.d. in the dihedral angle between two l.s. planes) are estimated using the full covariance matrix. The cell e.s.d.'s are taken into account individually in the estimation of e.s.d.'s in distances, angles and torsion angles; correlations between e.s.d.'s in cell parameters are only used when they are defined by crystal symmetry. An approximate (isotropic) treatment of cell e.s.d.'s is used for estimating e.s.d.'s involving l.s. planes.
Refinement. Refinement of *F*^2^ against ALL reflections. The weighted *R*-factor *wR* and goodness of fit *S* are based on *F*^2^, conventional *R*-factors *R* are based on *F*, with *F* set to zero for negative *F*^2^. The threshold expression of *F*^2^ > σ(*F*^2^) is used only for calculating *R*-factors(gt) *etc*. and is not relevant to the choice of reflections for refinement. *R*-factors based on *F*^2^ are statistically about twice as large as those based on *F*, and *R*- factors based on ALL data will be even larger.

### Fractional atomic coordinates and isotropic or equivalent isotropic displacement parameters (Å^2^)

**Table:**

	*x*	*y*	*z*	*U*_iso_*/*U*_eq_	
C1	0.4972 (3)	0.8406 (3)	0.1955 (2)	0.0309 (5)	
H1	0.3863	0.8330	0.2844	0.037*	
C2	0.5378 (3)	0.7784 (2)	0.0396 (2)	0.0250 (4)	
C3	0.7038 (3)	0.7881 (3)	−0.0919 (2)	0.0312 (4)	
H3	0.7369	0.7485	−0.1993	0.037*	
C4	0.8196 (3)	0.8579 (3)	−0.0606 (3)	0.0367 (5)	
H4	0.9326	0.8647	−0.1468	0.044*	
C5	0.7665 (3)	0.9175 (2)	0.0990 (3)	0.0313 (4)	
H5	0.8457	0.9643	0.1180	0.038*	
C6	0.4027 (3)	0.7086 (2)	0.0214 (2)	0.0253 (4)	
Mn1	0.5000	1.0000	0.5000	0.02565 (17)	
N1	0.6059 (2)	0.9108 (2)	0.22757 (19)	0.0291 (4)	
N2	0.2536 (2)	0.6822 (2)	0.1516 (2)	0.0316 (4)	
N3	0.1654 (2)	0.6214 (2)	0.0825 (2)	0.0345 (4)	
N4	0.2572 (2)	0.6120 (2)	−0.0813 (2)	0.0335 (4)	
N5	0.4088 (2)	0.6669 (2)	−0.12397 (19)	0.0293 (4)	
O1	0.4248 (2)	1.25174 (18)	0.44974 (18)	0.0465 (4)	
H1B	0.3486	1.3288	0.5213	0.056*	
H1A	0.4587	1.2950	0.3548	0.056*	
O2	0.79389 (19)	0.99831 (18)	0.44105 (18)	0.0364 (4)	
H2A	0.8730	0.9173	0.4589	0.044*	
H2B	0.8229	1.0820	0.4526	0.044*	
O3	0.0823 (2)	0.75323 (18)	0.49811 (18)	0.0372 (4)	
H3B	0.0942	0.6696	0.5562	0.045*	
H3A	0.1346	0.7196	0.3958	0.045*	
O4	0.1519 (2)	0.48719 (18)	0.69383 (17)	0.0359 (4)	
H4B	0.0608	0.4519	0.7598	0.043*	
H4A	0.1855	0.5271	0.7575	0.043*	

### Atomic displacement parameters (Å^2^)

**Table:**

	*U*^11^	*U*^22^	*U*^33^	*U*^12^	*U*^13^	*U*^23^
C1	0.0302 (10)	0.0403 (11)	0.0217 (10)	−0.0155 (9)	−0.0058 (8)	−0.0023 (8)
C2	0.0279 (10)	0.0230 (9)	0.0239 (10)	−0.0064 (8)	−0.0108 (8)	−0.0002 (7)
C3	0.0317 (10)	0.0378 (11)	0.0216 (10)	−0.0100 (9)	−0.0071 (8)	−0.0061 (8)
C4	0.0286 (10)	0.0506 (13)	0.0277 (11)	−0.0162 (10)	−0.0043 (8)	−0.0034 (9)
C5	0.0277 (10)	0.0374 (11)	0.0320 (11)	−0.0116 (8)	−0.0134 (8)	−0.0010 (9)
C6	0.0292 (10)	0.0226 (9)	0.0234 (10)	−0.0065 (8)	−0.0104 (8)	−0.0011 (7)
Mn1	0.0264 (2)	0.0299 (3)	0.0210 (2)	−0.00911 (17)	−0.00862 (17)	−0.00332 (16)
N1	0.0311 (9)	0.0351 (9)	0.0230 (8)	−0.0125 (7)	−0.0105 (7)	−0.0017 (7)
N2	0.0307 (9)	0.0386 (10)	0.0264 (9)	−0.0142 (7)	−0.0091 (7)	−0.0018 (7)
N3	0.0341 (9)	0.0405 (10)	0.0320 (9)	−0.0165 (8)	−0.0122 (7)	−0.0018 (8)
N4	0.0370 (9)	0.0370 (10)	0.0314 (9)	−0.0153 (8)	−0.0152 (8)	−0.0008 (7)
N5	0.0348 (9)	0.0320 (9)	0.0237 (9)	−0.0141 (7)	−0.0109 (7)	−0.0014 (7)
O1	0.0623 (10)	0.0321 (8)	0.0252 (8)	−0.0059 (7)	−0.0044 (7)	0.0003 (6)
O2	0.0302 (7)	0.0385 (8)	0.0429 (9)	−0.0084 (6)	−0.0166 (6)	−0.0071 (6)
O3	0.0407 (8)	0.0377 (8)	0.0292 (8)	−0.0113 (7)	−0.0100 (6)	−0.0011 (6)
O4	0.0401 (8)	0.0413 (8)	0.0273 (8)	−0.0177 (7)	−0.0094 (6)	−0.0044 (6)

### Geometric parameters (Å, º)

**Table:**

C1—N1	1.337 (3)	Mn1—O2^i^	2.222 (3)
C1—C2	1.382 (3)	Mn1—O2	2.222 (3)
C1—H1	0.9300	Mn1—N1	2.290 (3)
C2—C3	1.383 (3)	Mn1—N1^i^	2.290 (3)
C2—C6	1.468 (3)	N2—N3	1.342 (2)
C3—C4	1.382 (3)	N3—N4	1.309 (3)
C3—H3	0.9300	N4—N5	1.349 (3)
C4—C5	1.377 (3)	O1—H1B	0.8500
C4—H4	0.9300	O1—H1A	0.8500
C5—N1	1.336 (3)	O2—H2A	0.8500
C5—H5	0.9300	O2—H2B	0.8501
C6—N5	1.331 (3)	O3—H3B	0.8500
C6—N2	1.338 (3)	O3—H3A	0.8501
Mn1—O1	2.132 (2)	O4—H4B	0.8500
Mn1—O1^i^	2.132 (2)	O4—H4A	0.8501
			
N1—C1—C2	124.70 (17)	O1—Mn1—N1	95.02 (7)
N1—C1—H1	117.6	O1^i^—Mn1—N1	84.98 (7)
C2—C1—H1	117.6	O2^i^—Mn1—N1	92.50 (6)
C1—C2—C3	117.48 (18)	O2—Mn1—N1	87.50 (6)
C1—C2—C6	118.90 (17)	O1—Mn1—N1^i^	84.98 (7)
C3—C2—C6	123.61 (18)	O1^i^—Mn1—N1^i^	95.02 (7)
C4—C3—C2	118.66 (19)	O2^i^—Mn1—N1^i^	87.50 (5)
C4—C3—H3	120.7	O2—Mn1—N1^i^	92.50 (6)
C2—C3—H3	120.7	N1—Mn1—N1^i^	179.999 (1)
C5—C4—C3	119.62 (19)	C5—N1—C1	116.74 (18)
C5—C4—H4	120.2	C5—N1—Mn1	127.06 (13)
C3—C4—H4	120.2	C1—N1—Mn1	116.17 (13)
N1—C5—C4	122.78 (19)	C6—N2—N3	104.94 (17)
N1—C5—H5	118.6	N4—N3—N2	109.54 (17)
C4—C5—H5	118.6	N3—N4—N5	109.28 (15)
N5—C6—N2	111.27 (17)	C6—N5—N4	104.97 (15)
N5—C6—C2	125.30 (17)	Mn1—O1—H1B	126.3
N2—C6—C2	123.42 (17)	Mn1—O1—H1A	127.6
O1—Mn1—O1^i^	180.0	H1B—O1—H1A	106.1
O1—Mn1—O2^i^	88.59 (7)	Mn1—O2—H2A	122.5
O1^i^—Mn1—O2^i^	91.41 (7)	Mn1—O2—H2B	123.2
O1—Mn1—O2	91.41 (7)	H2A—O2—H2B	106.1
O1^i^—Mn1—O2	88.59 (7)	H3B—O3—H3A	106.7
O2^i^—Mn1—O2	180.000 (1)	H4B—O4—H4A	105.2

Symmetry code: (i) −*x*+1, −*y*+2, −*z*+1.

### Hydrogen-bond geometry (Å, º)

**Table:**

*D*—H···*A*	*D*—H	H···*A*	*D*···*A*	*D*—H···*A*
O1—H1*B*···O4^ii^	0.85	1.94	2.783 (3)	172
O1—H1*A*···N5^iii^	0.85	1.91	2.731 (3)	163
O2—H2*A*···O3^iv^	0.85	1.99	2.836 (3)	171
O2—H2*B*···O3^i^	0.85	1.96	2.800 (3)	169
O3—H3*B*···O4	0.85	1.96	2.803 (3)	171
O3—H3*A*···N2	0.85	1.96	2.797 (3)	170
O4—H4*B*···N3^v^	0.85	2.03	2.878 (3)	177
O4—H4*A*···N4^vi^	0.85	2.00	2.849 (3)	176

Symmetry codes: (i) −*x*+1, −*y*+2, −*z*+1; (ii) *x*, *y*+1, *z*; (iii) −*x*+1, −*y*+2, −*z*; (iv) *x*+1, *y*, *z*; (v) −*x*, −*y*+1, −*z*+1; (vi) *x*, *y*, *z*+1.
